# Canine Histiocytic and Hemophagocytic Histiocytic Sarcomas Display *KRAS* and Extensive *PTPN11*/SHP2 Mutations and Respond In Vitro to MEK Inhibition by Cobimetinib

**DOI:** 10.3390/genes15081050

**Published:** 2024-08-09

**Authors:** Ya-Ting Yang, Alexander I. Engleberg, Ishana Kapoor, Keita Kitagawa, Sara A. Hilburger, Tuddow Thaiwong-Nebelung, Vilma Yuzbasiyan-Gurkan

**Affiliations:** 1 Department of Microbiology, Genetics and Immunology, Michigan State University, East Lansing, MI 48824, USA; yangyat1@msu.edu (Y.-T.Y.); engleber@msu.edu (A.I.E.); kapooris@msu.edu (I.K.); hilburg1@msu.edu (S.A.H.); 2 Department of Small Animal Clinical Sciences, Michigan State University, East Lansing, MI 48824, USA; 3 Comparative Medicine and Integrative Biology Program, College of Veterinary Medicine, Michigan State University, East Lansing, MI 48824, USA; kkitagawa@ufl.edu; 4 Veterinary Diagnostic Laboratory, College of Veterinary Medicine, Michigan State University, Lansing, MI 48910, USA; thaiwong@msu.edu

**Keywords:** histiocytic sarcoma, hemophagocytic histiocytic sarcoma, *PTPN11*, SHP2, *KRAS*, cobimetinib, MAPK pathway, MEK inhibitors, cancer genetics, myeloid tumors, comparative oncology

## Abstract

Histiocytic sarcoma (HS) is a rare and highly aggressive cancer in humans and dogs. In dogs, it has a high prevalence in certain breeds, such as Bernese mountain dogs (BMDs) and flat-coated retrievers. Hemophagocytic histiocytic sarcoma (HHS) is a unique form of HS that presents with erythrophagocytosis. Due to its rareness, the study of HHS is very limited, and mutations in canine HHS patients have not been studied to date. In previous work, our research group identified two major *PTPN11*/SHP2 driver mutations, E76K and G503V, in HS in dogs. Here, we report additional mutations located in exon 3 of *PTPN11*/SHP2 in both HS and HHS cases, further supporting that this area is a mutational hotspot in dogs and that mutations in tumors and liquid biopsies should be evaluated utilizing comprehensive methods such as Sanger and NextGen sequencing. The overall prevalence of *PTPN11*/SHP2 mutations was 55.8% in HS and 46.2% in HHS. In addition, we identified mutations in *KRAS*, in about 3% of HS and 4% of HHS cases. These findings point to the shared molecular pathology of activation of the MAPK pathway in HS and HHS cases. We evaluated the efficacy of the highly specific MEK inhibitor, cobimetinib, in canine HS and HHS cell lines. We found that the IC_50_ values ranged from 74 to 372 nM, which are within the achievable and tolerable ranges for cobimetinib. This finding positions cobimetinib as a promising potential candidate for future canine clinical trials and enhances our understanding of the molecular defects in these challenging cancers.

## 1. Introduction

Histiocytic sarcoma (HS) is an aggressive and rare cancer, occurring in less than 1% of human and canine cancer patients. Due to the rarity of HS, the development of effective treatments is an ongoing challenge. In dogs, although rare overall, HS is common in Bernese mountain dogs (BMDs) and flat-coated retrievers (FCRs), where a genetic predisposition is documented [1,2]. HS is also overrepresented in Rottweilers and golden retrievers [3,4]. While several loci have been identified to contribute to the risk of HS in BMDs [5] and FCRs [6], causative predisposing mutations have been elusive so far.

In both dogs and humans, histiocytic disorders range from benign proliferative lesions to highly aggressive sarcomas. In dogs, these include localized benign cutaneous masses (histiocytomas), reactive cutaneous or systemic histiocytosis, and benign proliferative diseases, but also overt malignancies that can present as localized or highly disseminated aggressive lesions involving multiple major organs (histiocytic sarcomas). Additionally, an HS subtype, hemophagocytic histiocytic sarcoma (HHS), presents with marked erythrophagocytosis. While most canine HS cases show evidence of dendritic cell lineage (being positive for CD11a, CD11b, and CD11c), HHS cases display CD11d, indicating macrophage lineage. A series of studies have identified driver mutations in the MAPK pathway in histiocytic diseases in humans [7,8] and dogs [9,10] in recent years. The use of targeted small molecule inhibitors of the MAPK pathway has shown promising results in humans [8], with some studies showing prolonged clinical response [11]. In dogs, our team was the first to report MAPK pathway activating mutations in *PTPN11* gene encoding the protein SHP2, and *KRAS* in histiocytic sarcomas [9,10]. In addition, we were able to demonstrate the efficacy of trametinib and dasatinib, both inhibitors of the MAPK pathway, in canine HS cell lines and in mouse models of canine HS [12,13]. In addition, after evaluating the safety of trametinib in a phase I study of tumor bearing dogs [14], we have initiated a clinical trial evaluating the efficacy of the drug in HS canine patients, which is ongoing. Other groups, have since reported various mutations in *PTPN11*/SHP2 and *KRAS* in canine HS [15,16].

In order to evaluate responses to therapies and effectively employ tools such as liquid biopsies, it is important to appreciate the full spectrum of mutations in driver genes in tumors. In the current study, we report our findings of extensive variation in a mutational hotspot region of exon 3 of *PTPN11* in canine HS, including the frequency of mutations in *PTPN11*/SHP2 and *KRAS* in canine hemophagocytic histiocytic sarcoma (HHS). The frequency of driver mutations in HHS cases has not been previously studied. Furthermore, we evaluated the response of various HHS and HS cell lines to cobimetinib, a dual MEK1/MEK2 inhibitor approved by the FDA for use on advanced melanoma patients in 2015 [17], as cobimetinib has also been successfully used to treat human HS patients [7,11]. As the MEK proteins (mitogen-activated protein kinase (MAPK)/extracellular signal-regulated kinase 1 and 2) integrate the cellular response to many growth factors and activation of signals in the MAPK pathway, their inhibition has proven effective in the growth of various tumors. As an orally available, highly specific inhibitor, cobimetinib is a powerful anti-cancer agent, whether used alone or in combination with other drugs [18,19,20].

## 2. Materials and Methods

### 2.1. Animal Use Statement

All Tissue Samples and Cell Lines Used in This Study Were Obtained Using Protocols Approved or Deemed Exempt by the Michigan State University IACUC (AUF#08/15-127-00).

### 2.2. Histiocytic Sarcoma (HS) Samples

All cases of HS included in this study were diagnosed by histopathology and were confirmed positive for CD204 by immunohistochemistry (IHC). Samples were either derived from cases directly submitted to the Michigan State University Veterinary Diagnostic Laboratory (MSU-VDL) or were part of our MSU BMD DNA and Tissue Repository as frozen tissues, stored in small pieces at −80 °C. Each frozen tumor tissue sample was sectioned into two pieces, one embedded in OCT and later transferred to paraffin and processed for histopathology, including IHC, and the other section used for DNA extraction. In total, 129 HS cases were analyzed, among which 96 were also included in a previous study [10].

### 2.3. Hemophagocytic Histiocytic Sarcoma (HHS) Samples

Fourteen HHS frozen BMD tumor tissue samples from the MSU BMD Tissue and DNA Repository and 12 cases from various breeds archived at MSU-VDL were included in the study. All samples were confirmed by histopathology by a board-certified pathologist (TTN) and were positive for CD11d expression by IHC.

### 2.4. Cell Lines

The cell lines used in the study included two HS cell lines from Bernese mountain dogs (BD and OD) previously established in our laboratory [21] and one HHS cell line, DH82, obtained from ATCC (CRL-3590). Cells were maintained in RPMI 1640 medium (Life Technologies, Carlsbad, CA, USA; ThermoFisher Scientific Inc., Waltham, MA, USA), supplemented with 10% fetal bovine serum (FBS), 1% anti-anti, antibiotic/antifungal agent (Gibco), and 0.1% Gentamicin. All cell lines were incubated at 37 °C at 5% CO_2_.

### 2.5. Genomic DNA Extraction

Tumor genomic DNA was extracted from formalin-fixed paraffin-embedded (FFPE) tissue using 5 to 8 10-micron sections and a QIAamp DNA FFPE Tissue Kit (Qiagen, Germantown, MD, USA). For frozen tumor tissue samples, a DNeasy blood and tissue kit (Qiagen, Germantown, MD, USA) was used with an input of about 25 mg of tissue. Quantification of DNA was carried out using a Qubit dsDNA HS kit (ThermoFisher, Waltham, MA, USA) and a Qubit 2.0 fluorometer (ThermoFisher, Waltham, MA, USA).

### 2.6. Genotyping using the TaqMan Custom SNP Genotyping Assay

Genotyping was performed using custom TaqMan SNP genotyping assays, listed in Table 1. Samples were genotyped for *PTPN11*/SHP2 G503V and KRAS Q61H, as described earlier [10]. For each TaqMan genotyping assay run, 5 μL of each tumor DNA sample, at 2 ng/μL, was mixed with 0.5 μL of the 20X TaqMan custom SNP Assay and 5 μL of the TaqMan Genotyping Master Mix (ThermoFisher, Waltham, MA, USA) in a 96-well plate. Two no-template negative controls with DNase-free water and three positive controls with known genotypes, previously confirmed by Sanger sequencing, were included in each run. The assays were run using a QuantStudio 3 (ThermoFisher, Waltham, MA, USA) real-time PCR machine, following the manufacturer’s recommended cycling conditions. All samples were analyzed in duplicate. The assays and details of the mutations analyzed are presented in Table 1.

### 2.7. Genotyping by Sanger Sequencing

Sanger sequencing was performed on all HHS samples, as well as on samples from our previous study that are either wildtype for PTPN11^E76^ or were not previously genotyped. Amplicons containing known multiallelic variant sites in *PTPN11* and *KRAS* were sequenced using primers indicated in Table 2. After DNA was amplified and visualized on a gel, the PCR products were isolated using an Exo-CIP Rapid PCE Cleanup Kit (New England Biolabs, Ipswich, MA, USA) and submitted for Sanger sequencing to the MSU Genomics Core, where they were sequenced in both directions.

### 2.8. Determination of IC_50_ Values

Cells were seeded on flat-bottom 96-well culture plates (Alkali Scientific, Fort Lauderdale, FL, USA) at a density of 3000 cells/well. After 24 h, cell culture media was replaced by complete media with the compound at the given concentration, and cells were treated with compounds for 72 h, after which the MTS reagent was added. Cell viability was analyzed using a CellTiter 96 Aqueous Non-Radioactive Cell Proliferation Assay (Promega, Fitchburg, WI, USA) and determined by the amount of colored formazan dye produced by live cells. The absorbance of formazan dye was measured at a 490 nm wavelength, and IC_50_ values were calculated using Graph Pad Prism 9.2.0 (Graph Pad Software Inc., San Diego, CA, USA). Each IC_50_ data point was obtained in triplicate, and each assay was run in three biological replicates.

### 2.9. Analysis of Amino Acid Substitution Effects

The predicted effects of the resulting amino acid substitutions in *PTPN11*/SHP2 were determined in silico using AlphaMissense for the human protein [22] and PolyPhen2 for the dog [23]. There is 99.3% identity between the human and dog proteins (based on NP_002825.3 and A0A4D6PF96, respectively), with no difference in predicted protein structure. The only differences in amino acid residues occur at residue 449 and in three residues in the region that codes for the C-terminal tail. For AlphaMissense, a value of >0.564 indicates a likely pathogenic outcome. For PolyPhen2, a value >0.908 indicates a “probably damaging” outcome, and a value between 0.446 and 0.908 indicates a “possibly damaging” outcome [22,23].

### 2.10. Statistical Methods

Categorical age parameters between groups were analyzed by an unpaired *t*-test using Graph Pad Prism 9.2.0 (Graph Pad Software Inc., San Diego, CA, USA). The *p* values were determined by two-tailed analysis with significance set at <0.05. The normal distribution of the data was verified with the Shapiro–Wilk test module in Graph Pad Prism 9.2.0.

## 3. Results

### 3.1. PTPN11/SHP2 and KRAS Mutational Status in HS Cases

**Figure 1:**
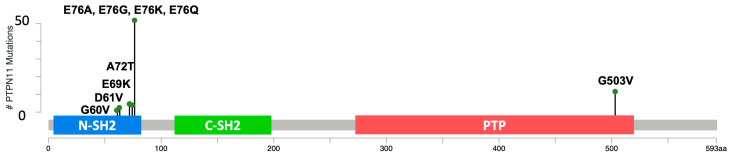
A lollipop plot of somatic mutations found in the HS samples diagramed on a *PTPN11*/SHP2 protein domain map, modeled after similar plots on cbioportal.org (accessed on 6 January 2024). The two Src homology domains are indicated with the label SH2, the N-terminal SH2 domain is indicated in blue, the C-terminal SH2 domain is indicated in green, and the phosphatase domain is indicated in red. The numbers along the x axis indicate the amino acid (aa) residues from the N to the C terminal of the SHP2 protein.

Sanger sequencing of 129 HS samples around the E76 residue in exon 3 of canine *PTPN11* revealed the presence of multiple mutations. Some of these mutations were previously reported, but additional mutations were found. Fourteen of the 96 samples previously found to be wildtype at E76 when tested with TaqMan genotyping, were found to carry other *PTPN11* exon 3 mutations when Sanger sequencing was used. The additional mutations within these samples included 1 G60V, 1 D61V, 2 E69K, 4 E76A, 3 E76G, 1 E76Q, and 1 A72T. The frequency of each mutation is presented in Table 3 below and schematically depicted in Figure 1. For the Sanger results, analysis of sequence chromatograms using IGV [24] was performed to determine the presence of variants. Heterozygosity was determined when a variant signal was at least 20% the strength of the wild-type signal at the same nucleotide. Figure S1 depicts representative chromatograms showing the presence of the *PTPN11*/SHP2 variants G60V, D61V, A72T, and E76K in BMD HS samples.

### 3.2. PTPN11/SHP2 and KRAS Mutational Status in HHS Cases

A total of 26 HHS cases were analyzed. Of the 26 cases, 14 were BMDs; other breeds included golden retrievers (*n* = 4), mixed breed (*n* = 3), and one dog each of the poodle, Labrador retriever, French bulldog, Welsh corgi, and rat terrier breeds. The average age at diagnosis was 7.7 ± 2.9 years. There were 12 males and 14 females. Among the 26 cases, 12 dogs carried either one *PTPN11*/SHP2, one KRAS, or one *PTPN11*/SHP2 and a KRAS mutation.

**Figure 2:**
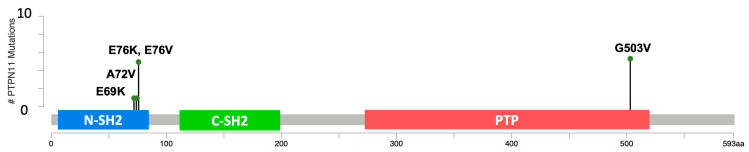
A lollipop plot of mutations found in the HHS samples diagramed on a *PTPN11*/SHP2 protein domain map, modeled after similar plots on cbioportal.org (accessed on 6 January 2024). The two Src homology domains are indicated with the label SH2, the N-terminal SH2 domain is indicated in blue, the C-terminal SH2 domain is indicated in green, and the phosphatase domain is indicated in red. The numbers along the x axis indicate the amino acid (aa) residues from the N to the C terminal of the SHP2 protein.

**Figure 3:**
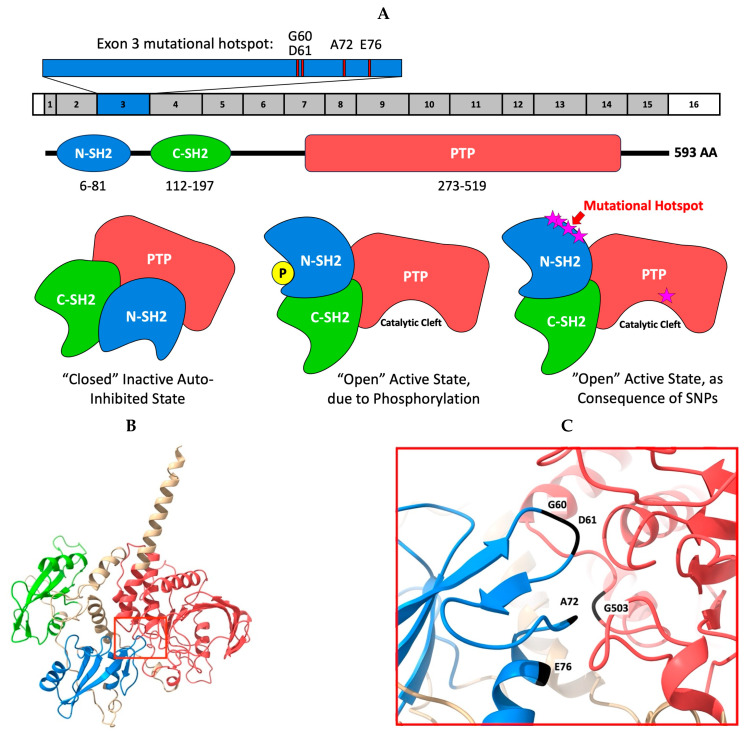
Cartoon and 3D model representation of the canine SHP2 protein. The protein transcript used is the 593 amino acid peptide (A0A4D6PF96). (**A**) A schematic domain map depicting the locations of the N- and C-terminal SRC homology 2, or SH2, domains (blue and green) and phosphatase, or PTP, domain (red), with depictions of the auto-inhibited inactive and “open” active conformations of the SHP2 protein. In its inactive state, the N-SH2 domain binds to the PTP domain around the PTP catalytic site. Mutations in the N-SH2 and PTP domains help to promote the “open” conformation. Pink stars represent SNPs that result in the promotion of the active state and increased catalytic activity. Additionally, an exon map of the SHP2 protein highlights the exon 3 mutational hotspot. (**B**) 3D model of the SHP2 protein depicting the auto-inhibited conformation. The catalytic cleft is bounded by a red box. (**C**) Zoomed in view of a portion (highlighted in panel (**B**)) of the cleft encompassing numerous known mutation sites, including the mutations presented here. The residues G60, D61, A72, and E76 are located in the N-SH2 domain (E69 not captured in panel (**C**)). The G503 residue is located in the PTP domain. Cartoon inspired by Chen et al. [25]. 3D protein models were generated from AlphaFold [26,27] and UCSF ChimeraX [28].

The prevalence of *PTPN11*/SHP2 mutations in HHS cases was found to be 46.2% in the 26 cases analyzed. As summarized in Table 4, four HHS cases (15.4%) were positive for the E76K variant, and five (19.2%) were positive for the G503V variant. Other mutations in *PTPN11*/SHP2 included E69K (*n* = 1, 3.8%), E76V (*n* = 1, 3.8%), and A72V (*n* = 1, 3.8%). In the current study, we found one dog (3.8%) with a KRAS mutation, the G12D variant. However, we did not identify any cases with the KRAS^Q61H^ mutation in the current study. The prevalence of mutations in HHS cases is illustrated in Figure 2 and summarized in Table 5 (*PTPN11*/SHP2) and Table 6 (KRAS). The variants are noted on a three-dimensional model of the protein illustrated in Figure 3.

### 3.3. Age and Sex Status in HS and HHS Cases

The average age at diagnosis of HS in the 129 BMDs was 8.4 ± 2.1 years old, including 64 males and 65 females. The average age of BMDs with identified mutations was 8.1 ± 2.2 years.

In the HHS group, the average age at diagnosis was 6.6 ± 3.0 years old in BMDs, whereas the average age at diagnosis of other breeds was 8.9 ± 2.3, which is significantly higher (*p* < 0.05). Of the 26 HHS cases, there were 12 male and 14 female dogs.

### 3.4. Cobimetinib Inhibits HS and HHS Cell Growth

The MEK1/MEK2 inhibitor cobimetinib demonstrated effective inhibition of cell growth in the three cell lines tested. The IC_50_ values in 2 HS and 1 HHS cell lines are summarized in Table 7. The IC_50_ values are 74, 91, and 372 nM, respectively, for the BD, OD, and DH82 cell lines. The values for Cmax, the maximum concentration of cobimetinib achievable in humans [18] and dogs [29], are presented in the last column. The IC_50_ values for cobimetinib in all three cell lines are well below the Cmax of 1640 nM in dogs, indicating the high potential for use of cobimetinib in the clinic. The three cell lines have distinct mutational profiles, as presented in Table 7, and yet their IC_50_ values are all well below the achievable concentration of cobimetinib in dogs.

## 4. Discussion

Mutations in *PTPN11*/SHP2 have been previously documented in human and canine HS [7,10,15]. Our current study expands the spectrum of mutations identified in canine HS and presents the first report of *PTPN11*/SHP2 and KRAS mutations in canine HHS patients. With the additional mutations we have identified in *PTPN11*/SHP2, the frequency of *PTPN11* mutations in canine HS is 55.8%, while that of KRAS is 3% in 129 HS cases. Among these cases, 74 BMDs carried either a *PTPN11* or KRAS mutation, constituting 56.9% of all HS cases. *PTPN11*/SHP2 mutation status in canine HHS has been limited in the literature to that of the DH82 cell line, which harbors the G503V allele, as we have reported [10]. The current study is the first to address the mutation status in an HHS patient cohort. Our findings demonstrate that somatic mutations in *PTPN11* are common in canine HHS, with an overall frequency of 46.2% in 26 cases, with a frequency of 64.3% in BMDs (9/14) and 25% in other breeds (3/12). The prevalence of *PTPN11* mutations in HHS is similar to our findings in the HS patient cohort. Interestingly, BMDs displayed a significantly higher rate of *PTPN11* mutations, 64.3% vs. 25%, than dogs of other breeds. In addition, our findings include the identification of two novel mutations not previously reported in canine HS, D61V and A72T. In HHS samples, two novel mutations, A72V and E76V, were identified in our study.

As depicted in Figure 3, the SHP2 protein is composed of three main functional domains: two N-terminal Src-homology 2 domains (N-SH2 and C-SH2) preceding a catalytic PTP domain, and a C-terminal tail with two potential phosphorylation sites [25,30]. In its inactive state, the SHP2 protein is in a “closed” state, with the N-SH2 domain, encoded by exons 2 and 3 of *PTPN11*, binding to the phosphatase (PTP) domain (encoded by exons 7–13), blocking the catalytic site [25,30]. All of the mutations reported are in residues located in and around the catalytic cleft [25,30]. Residues G60, D61, A72, E76, and G503 are involved in forming hydrogen bonds to maintain the protein in the closed conformation [30], with the mutations being predicted to disrupt the closed state, making the substrates accessible to the phosphatase domain and increasing phosphatase activity. A full table of our identified *PTPN11* variants and their predicted consequences is found in Table S3.

As seen in Table 4, the E69K variant is predicted to be deleterious by AlphaMissense, yet PolyPhen2 predicts a tolerated substitution in both the dog and human proteins. While in silico predictions are conflicting, a previous in vivo study demonstrated that the E69K variant leads to an increase in SHP2 phosphatase activity compared to wild-type [31].

Our findings point to *PTPN11* exon 3 as a hotspot of mutations in HS and HHS in canines, as depicted in Figure 1 and Figure 2. One of the difficulties of screening through the presence of variants in the major hotspots is to distinguish variants in multiallelic hotspots, potentially harboring one of many single-nucleotide variants, in close proximity to each other. This aspect is problematic when using TaqMan genotyping assays or droplet PCR methods [32] to detect variants. This is especially true for the PTPN11^E76^ position, where droplet PCR methods as reported, as well as TaqMan genotyping, would miss a significant number of variants [16]. Using Sanger sequencing, we could distinguish PTPN11^E76K^, PTPN11^E76A^, PTPN11^E76G^, and PTPN11^E76Q^. In addition, we were able to identify mutations in nearby spots on exon 3, including PTPN^E69K^, PTPN11^A72V^, PTPN11^A72T^, PTPN11^G60V^, and PTPN11^D61V^ mutations. Among the 85 samples that were previously WT for PTPN11^E76K^ with the TaqMan genotyping, we identified 17 additional PTPN11 variants, which account for 13% of the 129 HS patient cohort. This population would have been missed if Sanger sequencing had not been used. In addition, 3 out of 26 HHS cases presented with a PTPN11 variant other than E76K, which accounts for 11.5% of all HHS cases. Therefore, for HS and HHS cases, using Sanger sequencing or targeted NextGen sequencing is recommended for the detection of mutations in tumors.

In HHS patients, our data shows the frequency of KRAS^Q61H^ mutations to be 4%, very similar to our previous findings of 3.1% in 96 HS dogs [10]. Clearly, the *PTPN11* and KRAS mutation frequencies in HS and HHS are similar. The similarity of mutation frequencies further indicates that regardless of cell lineage, the molecular pathology is similar, implying that effective treatments can be developed for both HS and HHS targeting the same altered pathways. While the frequency of the KRAS^Q61H^ mutation is low, it has an activating role in the same pathway as *PTPN11*/SHP2, specifically the MAPK pathways. The frequency of KRAS mutations varies across cancer types, being particularly high in pancreatic, colorectal, and lung cancers. In one study of human HS patients, 4 of 28 cases reported (14%) [7] carried KRAS mutations, including one of each of KRAS^G12D^ and KRAS^G13D^, and two KRAS^A146T^ mutations. In another paper on human histiocytic neoplasms, Diamond et al. reported 3 out of 18 patients (17%) having KRAS mutations, including KRAS^G12R^, KRAS^G13C^, and KRAS^R149G^ [33]. Interestingly, in a recent case report, a positive response to treatment with cobimatinib was observed in a human Rosai–Dorfman disease patient with the KRAS^G12R^ mutation [34]. Therefore, the identification of even rare mutations may be consequential for the treatment of patients. Other genes in MAPK may also contribute to tumorigenesis in HS and HHS. We are continuing to characterize alterations across key pathways in canine HS and HHS tumor samples.

Hemophagocytic histiocytic sarcoma is a rare but highly aggressive type of histiocytic sarcoma, with most cases presenting with anemia and splenomegaly [35]. In dogs, HHS cases have been reported in various breeds, including Bernese mountain dogs [36], flat-coated retrievers [35,37,38], English setters [39], Cavalier King Charles Spaniel dogs [40], greyhounds [41], golden retrievers [36], Rottweilers [36], Labrador retrievers [36], Schnauzers [36], and mixed breeds [36]. In humans, reported cases of HHS are very rare, with only a few documented case reports of splenic HS patients presenting with additional hemophagocytic features [42,43]. The rarity of cases poses additional challenges for enhancing our understanding of the disease. Furthermore, full immunohistochemical characterization with CD11d is not universally available [38]. Current treatments are lacking in efficacy for both HS and HHS. In a case report, one canine patient was reported to show clinical benefit from the combination treatment of doxorubicin and zoledronate [41]. In the study of lomustine in 10 flat-coated retrievers with HHS, Elliot et al. reported no significant improvement in clinical outcomes [38]. The mean survival time for HHS has been reported to be less than 2 months [36]. Clearly, novel and more effective therapeutic strategies are needed for HS and HHS patients.

The establishment of tumor cell lines is a critical step in drug screening. So far, only one cell line, DH82, derived from an HHS patient, is commercially available from ATCC [44,45]. Identifying and understanding the key mutations in canine HS and HHS will aid clinicians and researchers in developing more effective therapeutic strategies, discovering potential target therapies, and identifying patients that would benefit from personalized treatments. Previous studies in our laboratory have identified trametinib and dasatinib as effective against HS in in vitro and in vivo preclinical studies [12,13]. Trametinib is a MEK inhibitor, and dasatinib inhibits multiple kinases, including SRC family kinases in the MAPK signaling pathway. In our current study, we evaluate the use of the MEK1/MEK2 inhibitor, cobimetinib, on HS and HHS cell lines. We found the IC_50_ values in two HS and one HHS cell lines to be 74, 91, and 372 nM, respectively. These concentrations are well below the reported plasma achievable concentration (Cmax) in human patients treated with 60 mg and 100 mg doses, with the Cmax reported as 231 nM (273 ng/mL) and 550 nM (649 ng/mL), respectively [18], and within tolerated limits. In dogs, the Cmax was reported as 1.6 μM in dogs receiving 5 mg/kg [29], which strongly indicates the potential of using cobimetinib in canine HS and HHS patients.

In a recent review of human cases, targeted therapy with a MEK or BRAF inhibitor or dual MEK/BRAF inhibitors used on histiocytic neoplasms has shown benefit to patients with and without a BRAF^V600E^ variant, with treatments lasting over 6 months [46]. Another study reported effective treatment with trametinib for the Erdheim–Chester disease (ECD) and Rosai–Dorfman disease (RDD) subgroups of human histiocytic disorders with mutations in BRAF or other MAPK pathway-associated genes [11]. A human clinical trial (NCT02649972) of cobimetinib has recently been carried out, and a preliminary report indicates a positive response to treatment [47] in patients with histiocytic neoplasms [33]. Therefore, further studies evaluating a range of MAPK inhibitors, including cobimetinib, are warranted in dogs with HS and HHS.

## 5. Conclusions

Mutations in *PTPN11*/SHP2 are frequent in canine HS and HHS. Mutations in KRAS are found in 3–4% of cases. As both sets of mutations point to the activation of the MAPK pathway, rational treatment approaches, including targeting of this pathway, provide hope for the development of effective therapies. Our study adds cobimetinib to the potential arsenal of agents we can employ in future canine clinical trials. These findings further support the relevance of studying spontaneous canine cancers for human malignancies.

## Figures and Tables

**Table 1:** Custom-TaqMan SNP primers used in genotyping.

Gene (Protein) Name	Mutation	Assay ID	Genomic Coordinates of Mutations *	Location in cDNA
*PTPN11* (SHP2)	E76K	ANT2AD7 (Lot P180405-000 B02)	Chr26: 10,340,007	c.440G>A (XM_038575149.1)
	G503V	ANDJ2M3 (Lot P180403-001 E05)	Chr26: 10,377,939	c.1,508G>T (XM_038575149.1)
*KRAS*	Q61H	AN47YG4 (Lot P180307-008 A08)	Chr27: 24,263,793	c.183A>C (XM_038577089.1)

* CanFam4 reference genome used for genomic coordinates.

**Table 2:** Primers for amplicons of *PTPN11* and *KRAS* used for Sanger sequencing.

Gene Name	Forward Primer Sequence (5′ -> 3′)	Reverse Primer Sequence (5′ -> 3′)	Amplicon Size	Amplicon Coordinates *
*PTPN11* (exon 3)	GGAAAGGAGCCAGGCAACAA	TGGCATGGAAGAGGTGCATT	395 bp	chr26: 10339752+10340146
*KRAS* (exon 2)	AAAGGTGTTGATAGAGTGGGT	AGCCAATGGAACCCAAGTACA	380 bp	chr27: 24279858-24280237

* CanFam4 reference genome used for genomic coordinates.

**Table 3:** *PTPN11*/SHP2 Mutations in Canine HS Samples in BMDs.

Frequency of All Mutations in *PTPN11*/SHP2	55.8% (72/129)								
Frequency of *PTPN11*/SHP2 Mutations in 2 Exons	Exon 3 47.3% (61/129)								Exon 13 8.5% (11/129)
*PTPN11*/SHP2 Mutations	G60V	D61V	E69K	E76A	E76G	E76K	E76Q	A72T	G503V
Number of dogs	2	1	4	4	3	44	1	2	11
Frequency in 129 HS cases (current study)	1.6%	0.8%	3.1%	3.1%	2.3%	34.1%	0.8%	1.6%	8.5%
Status: New or previously reported [reference] (species)	[15] (dog)	New	[15] (human)	[16] (dog)	[15] (dog)	[9] (dog)	[16] (dog)	New	[10] (dog)
Reported in human cancers: (per cBioportal)	yes	no	yes	yes	no	yes	no	yes	yes
Amino Acid Substitution Effects:									
AlphaMissense (human)	1.000	0.999	0.998	0.998	0.997	1.000	0.997	0.998	1.000
PolyPhen2 (dog protein)	1.000	0.997	0.012	0.999	1.000	0.997	0.997	0.967	1.000

**Table 4:** *PTPN11*/SHP2 mutations in canine HHS samples in BMDs and other breeds.

Frequency of All Mutations in *PTPN11*/SHP2	Total Percentage: 46.2% (12/26 dogs)				
Frequency of *PTPN11*/SHP2 Mutations in 2 Exons	Exon 3 Frequency: 26.9% (7/26)				Exon 13 Frequency: 19.2% (5/26)
*PTPN11*/SHP2 Mutations	E69K	A72V	E76K	E76V	G503V
Number of dogs	1	1	4	1	5
Frequency in 26 HHS cases	3.8%	3.8%	15.4%	3.8%	19.2%
[reference] (species)	[15] (human)	New variant	[9] (dog)	New variant	[10] (dog)
Reported in human cancers: (per cBioportal)	yes	yes	yes	no	yes
Amino Acid Substitution Effects:					
AlphaMissense (human)	0.998	0.999	1.000	1.000	1.000
PolyPhen2 (dog protein)	0.012	0.962	0.997	0.998	1.000

**Table 5:** Frequency of tumor *PTPN11*/SHP2 mutations based on tumor type, breed, and age.

Groups	N	Avg Age	*PTPN11*/SHP2 Mutant	E76K	G503V	Other Variants
HS BMD	129	8.4	72 (56%)	44 (34%)	11 (9%)	17 (13%)
HHS all cases	26	7.7	12 (46%)	4 (15%)	5 (19%)	3 (12%)
HHS BMD	14	6.6	9 (64%)	4 (29%)	4 (29%)	1 (7%)
HHS other breeds	12	8.9	3 (25%)	0	1 (8%)	2 (17%)

**Table 6:** Frequency of tumor KRAS mutation status based on tumor type, breed, and age.

Groups	N	KRAS Mutant	Q61H	G12A	G12D
HS BMD	129	4 (3%)	3 (2%)	1 (0.8%)	0
HHS all cases	26	1 (4%)	0	0	1 (4%)
HHS BMD	14	1 (7%)	0	0	1 (7%)
HHS other breeds	12	0	0	0	0

**Table 7:** Mutational characteristics of the HS and HHS cell lines and IC_50_ values for cobimetinib.

	BD (HS)	OD (HS)	DH82 (HHS)	Cmax
Cobimetinib	74 nM	91 nM	372 nM	550 nM in humans [18] 1640 nM in dogs [29]
PTPN11^E76K^	Homozygous Mutant	Wild-Type	Wild-Type	
PTPN11^G503V^	Wild-Type	Heterozygous	Homozygous Mutant	

## Data Availability

All data reported are in the body and Supplemental Files of this manuscript.

## Supplementary Materials

The following supporting information can be downloaded at: https://www.mdpi.com/article/10.3390/genes15081050/s1: Figure S1: Sanger sequencing chromatograms depicting *PTPN11* variants; Table S1: Information on HS cases; Table S2: Information on HHS cases: Table S3: Predicted amino acid substitution effects of PTPN11 variants determined by AlphaMissense and PolyPhen2.
