# Supplementary material for: Canine Histiocytic and Hemophagocytic Histiocytic Sarcomas Display KRAS and Extensive PTPN11/SHP2 Mutations and Respond In Vitro to MEK Inhibition by Cobimetinib
Source: Genes (Basel). 2024 Aug 9;15(8):1050. doi: 10.3390/genes15081050 (PMC11353564; doi:10.3390/genes15081050)
Supplement: Supplementary file 1 [file genes-15-01050-s001.zip › Figure S1.pdf]

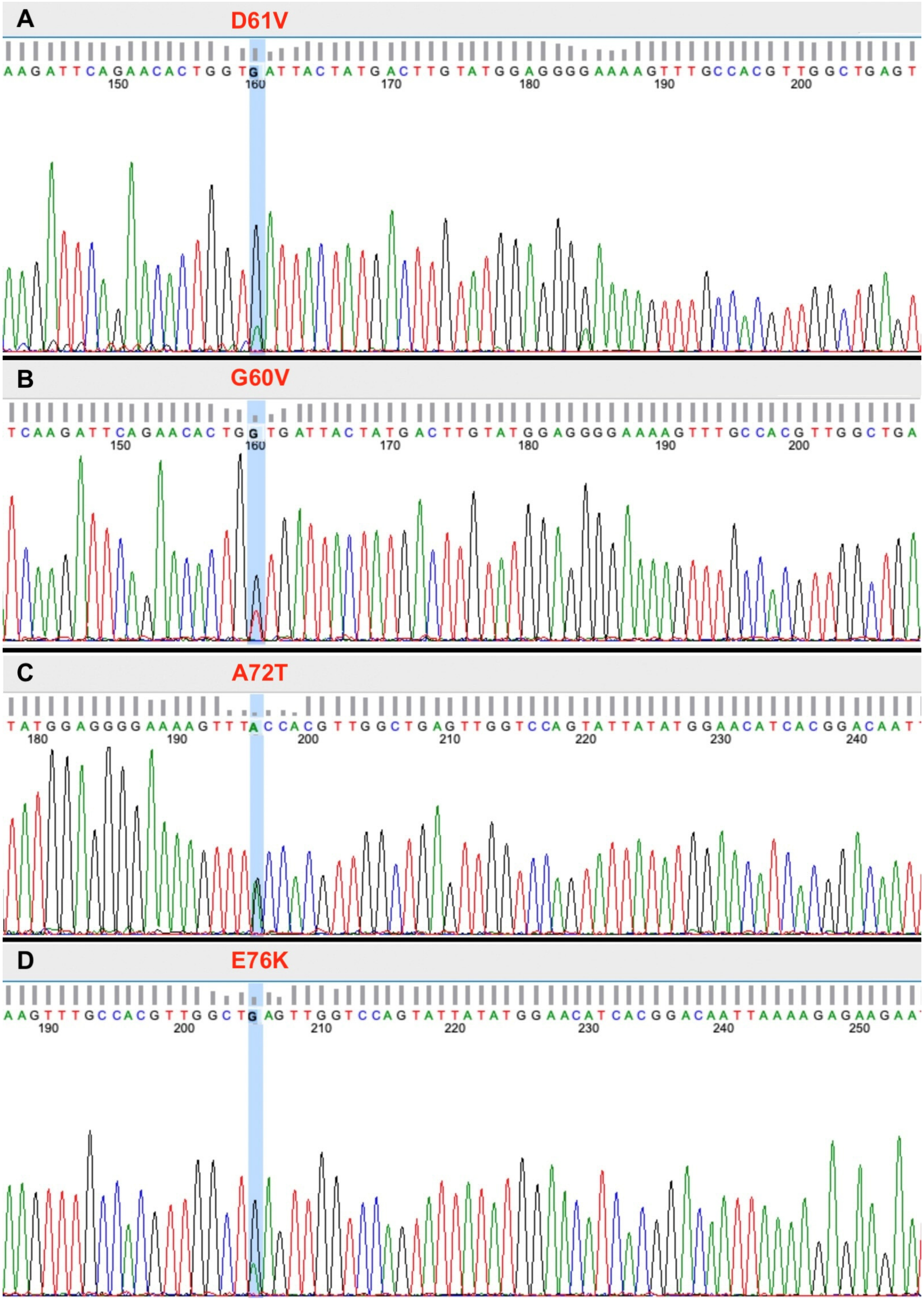

Figure S1: Sanger sequencing chromatograms depicting the A) D61V, B) G60V, C) A72T, and D) E76K PTPN11 variants. The polymorphisms of interest are highlighted in blue. A cutoff of >20% of the wild type signal was set to determine presence of variant alleles. Images produced using the IGV software package [21].
