# Supplementary material for: Canine Histiocytic and Hemophagocytic Histiocytic Sarcomas Display KRAS and Extensive PTPN11/SHP2 Mutations and Respond In Vitro to MEK Inhibition by Cobimetinib
Source: Genes (Basel). 2024 Aug 9;15(8):1050. doi: 10.3390/genes15081050 (PMC11353564; doi:10.3390/genes15081050)
Supplement: Supplementary file 1 [file genes-15-01050-s001.zip › Table S1.pdf]

Table S1. Information of HS cases

Label ID	Age (years)	Sex	PTPN11 mutations	KRAS mutations
1	10.1	M	E76K	WT
2	7.7	MN	G503V	WT
3	10.2	M	WT	WT
4	9.6	F	WT	WT
5	10.3	F	WT	WT
6	8.4	MN	WT	WT
7	8.7	F	E76G	WT
8	8.5	F	E76K	WT
9	9.2	F	WT	WT
10	8.2	M	E69K	WT
11	9.6	FS	WT	WT
12	6.9	F	E76A	WT
13	6.8	F	E76K	WT
14	7.7	F	WT	WT
15	5.9	M	WT	WT
16	10	MN	E76K	WT
17	4.6	M	E76K	WT
18	7.6	M	WT	WT
19	6.7	M	G503V	WT
20	7.7	FS	E76K	WT
21	5	MN	E76A	WT
22	9.9	F	WT	WT
23	8.3	M	WT	WT
24	2.6	M	G60V	WT
25	12.5	MN	WT	WT
26	9.1	F	E76K	WT
27	9.1	F	E76G	WT
28	10.3	F	WT	WT
29	9.8	F	WT	WT
30	8.4	M	WT	WT
31	10.7	M	G503V	WT
32	8.1	F	WT	Q61H
33	9.2	F	E76Q	WT
34	9	F	WT	WT
35	8.4	MN	E76A	WT

36	7	FS	WT	WT
37	10.5	FS	E76K	WT
38	7.5	M	E76K	WT
39	7.2	M	WT	WT
40	7.7	M	E76K	WT
41	6.8	FS	WT	WT
42	8.3	MN	E76K	WT
43	7.4	MN	WT	WT
44	8.8	M	E76K	WT
45	5.1	FS	G503V	WT
46	8.8	M	E76K	WT
47	8.8	M	E76K	WT
48	10.6	F	G60V	WT
49	7.9	F	E76K	WT
50	8.8	MN	E76G	WT
51	7.3	MN	E76K	Q61H
52	10.1	F	E76K	WT
53	9.1	MN	WT	WT
54	10.2	F	E76K	WT
55	9	M	E76K	WT
56	10.6	FS	WT	WT
57	8	FS	E76K	WT
58	10	F	WT	WT
59	6.6	F	WT	WT
60	7.2	F	WT	WT
61	8.3	M	WT	WT
62	10.4	FS	WT	WT
63	5.1	M	WT	WT
64	5.4	F	G503V	WT
65	9	MN	WT	WT
66	8.8	M	E76K	WT
67	9.6	MN	WT	WT
68	10.3	MN	G503V	WT
69	6.3	MN	WT	WT
70	4.6	FS	G503V	WT
71	12.4	FS	WT	WT
72	8	MN	WT	WT
73	4.7	FS	E76K	WT
74	7	M	A72T	WT

75	11	FS	E76K	WT
76	6.6	F	D61V	G12A
77	8.1	FS	E76A	WT
78	8.4	F	E69K	WT
79	8	FS	G503V	WT
80	7.1	FS	WT	WT
81	5.3	M	E76K	WT
82	7.1	M	WT	WT
83	7.5	MN	WT	WT
84	6.1	M	WT	WT
85	7	M	E76K	WT
86	10.8	F	E76K	WT
87	5	MN	E76K	WT
88	8	FS	E76K	WT
89	9.6	M	E76K	WT
90	9	F	E76K	WT
91	8.5	FS	WT	WT
92	8.5	FS	E76K	WT
93	12.2	F	WT	WT
94	10	M	G503V	Q61H
95	7.8	F	WT	WT
96	14	F	E76K	WT
97	5.7	F	E76K	WT
98	15	M	E76K	WT
99	N/A	F	WT	WT
100	6	MN	E76K	WT
101	16	F	E76K	WT
102	7.2	M	E76K	WT
103	8	F	WT	WT
104	9	M	WT	WT
105	9.4	M	E76K	WT
106	8.6	M	E76K	WT
107	5.8	M	G503V	WT
108	6.6	M	G503V	WT
109	9.3	M	WT	WT
110	5.7	F	E76K	WT
111	9.6	F	A72T	WT
112	6.2	F	WT	WT
113	10.3	M	WT	WT

114	4.3	M	WT	WT
115	6.1	F	E76K	WT
116	6.2	F	E76K	WT
117	9.3	M	WT	WT
118	7.9	F	WT	WT
119	6.7	F	WT	WT
120	8.7	M	WT	WT
121	11.3	F	E76K	WT
122	10.6	F	WT	WT
123	8.8	M	WT	WT
124	8.4	F	WT	WT
125	8.2	M	WT	WT
126	10.1	F	WT	WT
127	6.4	M	E69K	WT
128	10.5	F	E69K	WT
129	9.3	M	E76K	WT
