# Supplementary material for: Canine Histiocytic and Hemophagocytic Histiocytic Sarcomas Display KRAS and Extensive PTPN11/SHP2 Mutations and Respond In Vitro to MEK Inhibition by Cobimetinib
Source: Genes (Basel). 2024 Aug 9;15(8):1050. doi: 10.3390/genes15081050 (PMC11353564; doi:10.3390/genes15081050)
Supplement: Supplementary file 1 [file genes-15-01050-s001.zip › Table S2.pdf]

Table S2. Information of HSS cases

HHS Project ID	Sex	AGE (YRS)	PTPN11 exon 3 mutations	KRAS 12 results
1	M	7.2	G503V	WT
2	M	13.7	WT	WT
3	FS	5.1	G503V	WT
4	F	1.9	WT	G12D
5	MN	4.8	G503V	WT
6	MN	6.6	A72V	WT
7	M	7.2	E76K	WT
8	FS	7.2	G503V	WT
9	M	6.9	WT	WT
10	MN	4.6	WT	WT
11	M	6.1	WT	WT
12	F	2.7	E76K	WT
13	MN	9.4	E76K	WT
14	F	9.4	E76K	WT
15	F	9.8	E76V	WT
16	F	7	E69K	WT
17	F	9	WT	WT
18	M	9	G503V	WT
19	F	11	WT	WT
20	F	6.5	WT	WT
21	F	9	WT	WT
22	F	5	WT	WT
23	M	8	WT	WT
24	F	7	WT	WT
25	M	12	WT	WT
26	F	13	WT	WT
