# Supplementary material for: Canine Histiocytic and Hemophagocytic Histiocytic Sarcomas Display KRAS and Extensive PTPN11/SHP2 Mutations and Respond In Vitro to MEK Inhibition by Cobimetinib
Source: Genes (Basel). 2024 Aug 9;15(8):1050. doi: 10.3390/genes15081050 (PMC11353564; doi:10.3390/genes15081050)
Supplement: Supplementary file 1 [file genes-15-01050-s001.zip › Table S3.pdf]

Table S3. Predicted amino acid substitution effects of PTPN11 variants determined by AlphaMissense and PolyPhen2. AlphaMissense was run using the human protein (hg38). PolyPhen2 was run using the dog protein (canFam4). The two protein transcripts have a 99.3% identify, with no difference in predicted protein structure. For AlphaMissense, a value of >0.564 indicates a likely pathogenic outcome. For PolyPhen2, a value >0.908 indicates a "probably damaging" outcome, and a value between 0.446 and 0.908 indicates a "possibly damaging" outcome [19, 20].

**PTPN11 Variant**	**AlphaMissense Score (human protein) NP_002825.3**	**AlphaMissense Prediction**	**PolyPhen2 Score (dog protein) A0A4D6PF96**	**PolyPhen2 Prediction**
G60V	1.000	Likely pathogenic	1.000	Probably damaging
D61V	0.999	Likely pathogenic	0.997	Probably damaging
E69K	0.998	Likely pathogenic	0.012	Benign
A72T	0.998	Likely pathogenic	0.967	Probably damaging
A72V	0.999	Likely pathogenic	0.962	Probably damaging
E76A	0.998	Likely pathogenic	0.999	Probably damaging
E76G	0.997	Likely pathogenic	1.000	Probably damaging
E76K	1.000	Likely pathogenic	0.997	Probably damaging
E76Q	0.997	Likely pathogenic	0.997	Probably damaging
G503V	1.000	Likely pathogenic	1.000	Probably damaging
